# Post-translational modification of transcription factors: perspectives in vascular medicine

**DOI:** 10.1038/s41419-025-07832-5

**Published:** 2025-07-12

**Authors:** Elena Astanina, Elisa Setten, Francesco Boccalatte, Federico Bussolino, Valentina Comunanza

**Affiliations:** 1 Department of Oncology, University of Torino, 10060 Candiolo, TO Italy; 2 Candiolo Cancer Institute, FPO – IRCCS, 10060 Candiolo, TO Italy

**Keywords:** Vascular diseases, Transcription factors

## Abstract

In vascular diseases, the perturbation of key metabolic pathways is extensively studied and recognized as both a causal factor and a consequence in disease onset and progression. Similarly, the dysregulation of the transcriptional landscape within vascular unit components (endothelial and vascular smooth muscle cells) is a pivotal aspect of disease pathogenesis. Metabolite-induced post-translational modifications of transcription factors significantly modulate their functionality, leading to drastic changes in transcriptional outcomes. This review offers a new perspective by elucidating the link between metabolic alterations and metabolite-induced post-translational modifications of transcription factors integral to the pathogenesis of four paradigmatic vascular pathologies: atherosclerosis, diabetes, pulmonary hypertension and tumor angiogenesis.

## Facts

- Altered activities of transcription factors (TFs) in the vascular unit are a hallmark of vascular diseases.
- Post-translational modifications (PTMs) orchestrate different regulations of transcription factors, influencing their stability, bioavailability, localization and DNA binding.
- Alterations in metabolism and the subsequent fluctuations of metabolic intermediates exploited for PTMs of TFs represent a rapid and flexible mechanism to link metabolism-affecting pathogenic stressors with the transcriptional machinery.
- In vascular dysfunctions like atherosclerosis, diabetes, tumor angiogenesis and pulmonary arterial hypertension, metabolic pathways leading to the synthesis of lactate, amino sugars, NO and H_2_S are largely dysregulated.
- Lactylation, glycosylation, nitrosylation, and sulphydrylation are PTMs that paradigmatically contribute to the fine-tuning of the vascular transcriptional response.

## Open questions

- What is the impact of specific metabolite-derived PTMs targeting TFs on gene regulatory networks sustaining vascular diseases?
- What is the effect of TF dosage in diseases involving the vascular unit?
- Could it be possible in the near future to design therapeutic approaches for cardiovascular diseases based on the selective control of TF activities modified by specific PTMs?
- Could it be clinically relevant to create a pathogenic PTM signature for TFs involved in cardiovascular diseases?

## Introduction

Blood vessels supply oxygen and nutrients, remove catabolites, influence organ function during development and contribute to tissue responses under physiological and pathological conditions. Endothelial cells (ECs) and vascular smooth muscle cells (VSMCs) form the functional unit. This vascular unit is generally quiescent in healthy adults, but it is rapidly activated in response to physiologic changes or pathological hits and assumes different biological features sustained by specific transcriptional programs and epigenetic modifications.

Precise control of gene expression levels is essential for cell functions and the transcriptional response to microenvironmental changes has to start immediately and be temporally regulated to persist over time or be rapidly down-modulated.

Post-translational modifications (PTMs) can covalently modify proteins, like histones and transcription factors (TFs), through the addition of functional groups. Some PTMs are induced by small proteins (ubiquitin, the ubiquitin-like protein NEDD8, small ubiquitin-like modifier-SUMO) and regulate protein degradation or subcellular localization, while others originate from metabolic intermediates (Table 1). Emerging evidences indicate that PTMs deeply modify the regulatory role of TFs in determining genetic programs in vasculature.

**Table 1:** Most common post-translational modifications that target aminoacidic residues.

Source	Metabolite/Enzyme	Process Description (Origin/Type)
Acetic acid*	Acetyl-CoA	Oxidative decarboxylation of pyruvate
		β-oxidation of fatty acids
		Ketogenic aminoacids
ADP-ribose	ADP-ribosyl transferases	It catalyzes the transfer of one or more units of ADP-ribose from NAD^+^ to glutamate, or aspartate or serine residues of proteins
Butyric acid*	Butyryl-CoA	β-oxidation of fatty acids
Carbohydrates	Derivates of aldohexoses	Can form an O-glycosidic or N-glycosidic linkages respectively with threonine, tyrosine and serine residues and with asparagine residues
	Ribose, glucose, and glucose 6-phosphate	Non-enzymatic attachment to basic amino acids (lysine, arginine, histidine) to form fructosamines
Citrulline	Arginine deiminases	Hydrolysis of guanidino group of arginine to produce citrulline and ammonia
Crotonic/Glutaric acid*	Glutaryl-CoA/Crotonyl-CoA	Catabolism of lysine, hydroxylysine and tryptophan
Formic acid*	3′-Formylphosphate	5′-oxidation of deoxyribose in DNA
Hydrogen sulfide	Cystathionine β-synthase	Cysteine or homocysteine and 3-mercaptopyruvate
	Cystathionine gamma-lyase	Intestinal microbiota
	3-Mercaptopyruvate sulfurtransferase	
Isocyanic acid*	Anion cyanate	It forms a covalent adducts with nucleophilic groups of aminocids
Isoprenoids	Prenyl transferase	Farnesyl pyrophosphate (15-C) and geranylgeranyl pyrophosphate (20-C) isoprenoids are produced during the biosynthesis of cholesterol
	Geranylgeranyl transferase	
	Farnesyltransferase	
Lactic acid*	Lactate	Pyruvic acid generated by glycolysis is reduced to lactate by the NADH-dependent lactate dehydrogenase (in hypoxia)
Malonic acid*	Malonyl-CoA	Biotin- and ATP-dependent carboxylation of acetyl-CoA by the acetyl-CoA carboxylase
		It represents the first step of long chain-fatty acid synthesis
Methane	S-adenosylmethionine	Methyl donor to the side chain of lysine, arginine, histidine, asparagine and glutamine
		Methylation regulates the behavior of proteins including their functions, stability, transcription and cellular compartmentalization
Miristic acid	Miristoyl-CoA	Produced by fatty acid synthase complex
Monoamines	Transglutaminase	It catalyzes the crosslinking between the monoamines and glutamine residues of proteins
NEDD8*	NEDD8-activating enzyme E1	Protein neddylation is a multistep process
	NEDD8-conjuagating enzyme E2	
	Substrate-specific NEDD8-E3 ligases	
Nitric oxide	Nitric oxide synthases	Nitric oxide derives from arginine
		S-nitrosylation defines the covalent bond between NO and the sulfhydryl residue of cysteine
Palmitic acid	Palmitoyl-CoA acyltransferase	It catalyzes the formation of a thioester bond between a cysteine residue and palmitic acid
Phosphate	Serine/threonine and tyrosine kinases	Protein phosphorylation occurs at the side chain of serine, threonine, tyrosine, histidine, cysteine, glutamate, arginine, and lysine
Propionic acid*	Propionyl- CoA	β-oxidation of odd fatty acids
		Decarboxylation of succinate
		Catabolism of valine and isoleucine through the activity of branched-chain 2-keto acid dehydrogenase complex
		Catabolism of threonine which is dehydrated by the serine/threonine dehydratase to 2-ketobutyrate
		Synthesis of cysteine
		Intestinal microbiota
Succinic acid*	Succinyl CoA	Intermediate of Krebs cycle
		Intermediate of catabolism of isoleucine, methionine, threonine, valine
		Intermediate of catabolism of odd fatty acids
SUMO*	Small ubiquitin-related modifiers family	SUMOylation participates to the control of protein stability, cellular topology and transcription
Ubiquitin*	Ubiquitin-activating enzymes	Ubiquitin is targeted to lysine residues and polymerized by a multistep pathway
	Ubiquitin-conjugating enzymes	
	Ubiquitin ligases	

^*^PTMs targeting lysine residues.

Metabolism is a versatile and rapid tool that adapts to cellular behavior in response to microenvironmental changes. Therefore, intermediate metabolites or final products can serve as substrates or cofactors to induce PTMs, which modulate the components of the transcriptional machinery and influence their global behavior.

This review summarizes the current knowledge and provides additional insight on how cell metabolism, including cellular energy status, influences PTMs of master TFs involved in vascular behavior. We will also propose perspectives that link metabolic alterations to potential TF-PTMs influencing the behavior of blood vasculature in four paradigmatic pathological scenarios: atherosclerosis, diabetes, tumor angiogenesis and pulmonary arterial hypertension (PAH).

### PTMs involved in regulation of TF stability

TF levels are determined by a balance between biosynthesis and degradation, both regulated by cellular signals and specific PTMs that direct TFs to degradation pathways. The ubiquitin-proteasome system accounts for 80-90% of protein degradation. SUMOylation and NEEDylation also affect protein stability and, consequently, the availability of proteins for cellular processes. Ubiquitination removes TFs after they have performed their function or maintains steady levels of unstable TFs, which are continuously produced and degraded. It can be quantitatively controlled, leading to tunable TF degradation and, consequently, distinct TF levels.

### Activities of TFs are a function of their concentrations

**Fig. 1:**
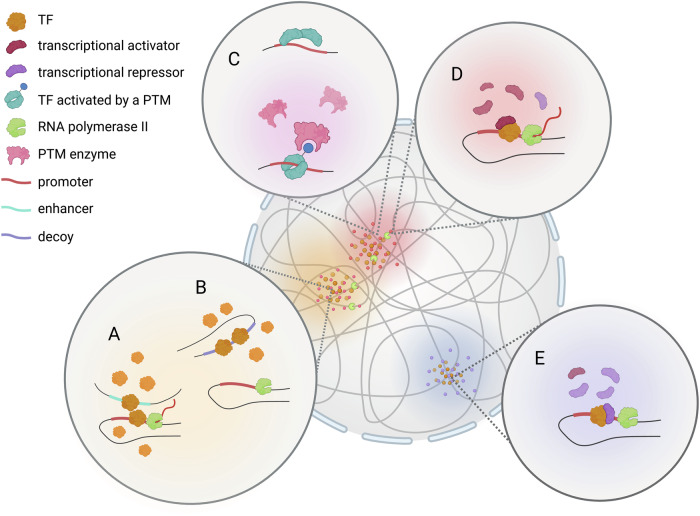
The law of mass action influences the regulation of the transcriptional landscape. Transcriptional hubs with transcription factors (TFs) and cofactors require phase-separated condensates around chromatin, facilitating biomolecule compartmentalization in a concentration-dependent manner. TFs bind distinct cis-regulatory elements (CREs) with varying affinity constants: high-affinity sites that perfectly match their motifs to initiate transcription (**A**) and high-affinity sites acting as decoys and sequestering TFs (**B**). The law of mass action influences enzyme-mediated PTMs (**C**) and the TF-DNA interactions by the recruitment of cofactors with activating or repressing roles (**D**, **E**). Created with Biorender.com.

The law of mass action governs numerous aspects of TFs dynamics and ultimately influences the regulation of the transcriptional landscape (Fig. 1) [1]. First, TFs recognize distinct cis-regulatory elements (CREs) — such as enhancers, silencers, and promoters — each characterized by different affinity constant. TFs not only bind to high-affinity sites that perfectly match their motifs to initiate gene transcription, but also interact with high-affinity sites acting as natural decoys that sequester TFs and prevent them from binding other sites. Additionally, TFs may bind to sites with lower affinity, but their cooperation with other weak binding sites can result in robust transcription [2]. Second, the law of mass action also influences enzyme-mediated PTMs, crucial for regulating TF stability, subcellular localization, activity and affinity for the binding sites [3]. Third, the final outcome of TF-DNA interactions in vivo is influenced by the formation of molecular complexes between TFs and cofactors with activating or repressing roles [4]. Fourth, recent findings suggest that transcriptional hubs with TFs and cofactors at genomic loci require phase-separation around chromatin that facilitates the functional compartmentalization of biomolecules in a concentration-dependent manner. Specific TFs have been described to regulate three-dimensional chromatin hubs, thus favoring gene expression and regulating cell identity and survival [5]. This phenomenon could be linked to TF titration and has been observed for PAX6 and SOX2 [6, 7]. Based on this, it is reasonable to hypothesize that the transcriptional landscape of a cell varies depending on the amount of TFs available [8–11]. Genetic studies show widespread phenotypic sensitivity to TF dosage. TFs are strongly enriched among genes whose loss-of-function variants cause haploinsufficient disease [12]. Minor variations in TF levels result in significant disease trait variations [13], in particular in aortic ECs [14]. Titration of HIF-1α through pO_2_-induced degradation triggers distinct transcriptional programs depending on hypoxia levels [9]. In embryonic stem cells, SOX2 dosage affects chromatin interaction and CRE selection. When SOX2 levels are high, CREs with high-affinity SOX2 binding sites become accessible to WNT effectors, preserving stemness. Conversely, low SOX2 levels reduce chromatin accessibility, promoting epiblast differentiation [10]. SOX9 dosage modulates CRE accessibility in a subset of pro-chondrogenic genes [11]. In cardiomyocytes, TBX5 dosages shape gene regulatory networks linked to congenital heart diseases [8]. Finally, NKX2-1 dosage threshold controls its oncogenic activities in several subtypes of lung cancer [15]. The pivotal role of TF titration has been also confirmed by a deep learning model that predicted how TWIST1 and SOX9 levels influence chromatin accessibility in facial progenitors. High-affinity, co-binding motifs at regulatory element centers buffer TF dosage changes and predict stable accessibility, while low-affinity lead to sensitivity with minimal baseline impact [16]. In living cells, chemical equilibrium between TF and DNA may be tuned by allosterism. For example, SOX2 binding to nucleosomes is mediated by cooperativity with OCT4 that alters nucleosome structure and promotes additional OCT4 and SOX2 binding to its internal sites [17]. Furthermore, these events alter chromatin conformation, regulating distant TF binding [18].

### The impact of PTM-mediated TF stability in vascular diseases

Our understanding of how gradual changes in TF dosage affect vascular disease pathogenesis is limited. However, alterations of protein stability and turnover by impairment of ubiquitin-proteasome pathway sustains insulin resistance, diabetes, and related metabolic dysfunctions [19]. Similarly, this system contributes to the development of atherosclerosis [20], PAH [21] and angiogenesis [22] by regulating vascular inflammation, metabolism, autophagy, oxidative stress, and cell growth and differentiation. Sporadic evidences directly link E3 ubiquitin ligase activity on TFs to vascular diseases. The most known pathway focuses on HIF- 1α subunit. The hydroxylation of proline residues enables ubiquitination via the von Hippel-Lindau E3 ligase complex [23]. In ECs, Parkin E3 ubiquitin ligase targets nuclear receptor ERRα, reducing levels of endothelial nitric oxide synthase (eNOS), an enzyme that is crucial for vascular fitness and is altered in vascular complications associated with diabetes and atherosclerosis [24]. The forkhead transcription repressor FoxO1, involved in insulin signaling, is inactivated in absence of the late endosome/lysosome-anchored MARCH3, an E3 ubiquitin ligase, leading to changes in the expression of genes involved in vascular permeability [25]. In a model of inflammatory vasculitis, the MDM2 ligase negatively regulates STAT3 by promoting its ubiquitination [26]. In calcific aortic valve disease, SMURF2 mediates ubiquitination of RUNX2, reducing vascular calcification [27]. The endothelial protocadherin FAT1 promotes YAP degradation by interaction with the E3 ligase MIB2, which stimulates YAP ubiquitination [28]. NRF2 forms a complex with KEAP1 E3 ligase and is degraded by the proteasome. Under oxidative stress, thiols in the cysteine residues in KEAP1 undergo modifications, decreasing KEAP1-mediated NRF2 ubiquitination and stabilizing NRF2 [29].

### PTMs targeting vascular transcription factors

**Fig. 2:**
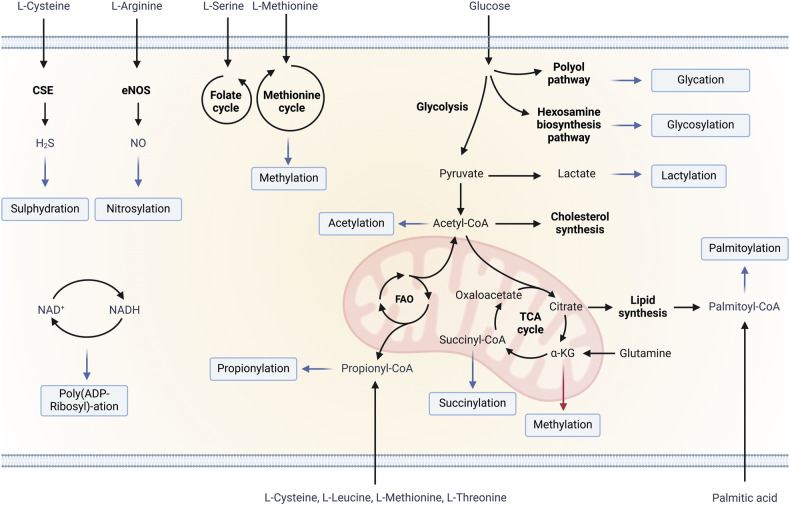
Schematic representation of key metabolite-induced post-translational modifications (PTMs) related to glucose and amino acid metabolism. Nitric oxide (NO) and hydrogen sulfide (H_2_S) derive from arginine and cysteine and are substrates for nitrosylation and sulphydration, respectively. S-adenosylmethionine is the methyl donor to the side chain of lysine, arginine, histidine, asparagine and glutamine for methylation. Poly (ADP-Ribosyl)-ation is characterized by the transfer of one or more units of ADP-ribose from NAD+ to glutamate, aspartate or serine residues. Carbohydrates can form an O-glycosidic or N-glycosidic linkages respectively with threonine, tyrosine and serine residues and with asparagine residues. Specific transferases and hydrolases catalyze this glycosylation process. Glycation can occur by non-enzymatic attachment of reducing monosaccharides like ribose, glucose, and glucose 6- phosphate to basic amino acids. In hypoxic conditions, pyruvic acid generated by glycolysis is reduced to lactate that can be substrate for lactylation. Acetyl-CoA is generated by the oxidative decarboxylation of pyruvate and by the β-oxidation of fatty acids (FAO) and is the source for acetylation. Propionyl-CoA, used for propionylation, is generated by the FAO, the catabolism of valine, isoleucine and threonine or during the synthesis of cysteine. Succinyl-CoA, the source for succinylation, is an intermediate of Krebs cycle. Palmitoyl-CoA acyltransferase catalyzes the formation of a thioester bond between a cysteine residue and palmitic acid, resulting in palmytoylation. Blue arrows indicate direct involvement of metabolites into the addition of PTMs, red arrow indicates the role of a metabolite as a cofactor for PTM-generating enzymes). Created with Biorender.com.

Metabolites represent a key source of molecules involved in protein PTMs and fine-tune protein functions in response to nutrient status and external cues. This regulatory mechanism becomes especially critical when the target proteins are TFs due to their holistic activity on cellular behavior. While classical PTMs like phosphorylation, methylation, and acetylation are well-studied, little is known about PTMs derived from primary or secondary metabolic intermediates (Table 2, Fig. 2). PTMs affect TF properties, as well as their spatial, temporal and tissue-specific activity. Therefore, it is intriguing to speculate that pathological alterations of substrate-product ratio of metabolic enzymatic reactions might have an impact on the availability of intermediates involved in PTMs of TFs and, consequently, modify the cellular transcriptional landscape. Additionally, three factors link metabolic alterations to TF activity and pathological phenotypes. First, the altered metabolic dynamics causes changes in concentration and distribution of metabolites used to modify TFs [30, 31]. Second, the half-lives of PTMs rely on the accessibility of enzymatic removal systems and catalytic properties. A third aspect to consider is the dynamic interplay between metabolic pathways, PTMs, and TFs. Metabolism influences the PTMs of TFs, which in turn regulate and modulate gene expression, including that of metabolic enzymes. Consequently, this intricate network of feedback and feed-forward loops adjusts the transcriptional landscape, influencing disease progression.

**Table 2:** Metabolism-dependent post-translational modifications (PTMs) of transcription factors (TFs) directly involved in endothelial (EC) and vascular smooth muscle cell (VSMC) functions^a^.

TF	Activities			PTM Type^d^
	**Formation of vascular bed in embryo and in adult life**	**EC fitness and pathological responses**	**Regulation of vascular smooth muscle cells**	
AP-1		Inflammatory response Vascular tone Thrombosis	Differentiation^f^	S-nitrosylation [141]
ATF4	Angiogenesis	Pathological angiogenesis Inflammatory response	Differentiation	No data available
CHOP			Proliferation Differentiation	No data available
COUP-TFII	Angiogenesis	Pathological angiogenesis		No data available
CREB	Angiogenesis	Pulmonary Hypertension	Differentiation Migration	Glycosylation [142] Propionylation [143]
DACH	Angiogenesis	Pathological angiogenesis		No data available
ETS	Vasculogenesis Angiogenesis	Inflammatory response Thrombosis Pathological angiogenesis	Proliferation	ADP-ribosylation [144]
FOXO1	Vasculogenesis Angiogenesis	Inflammatory response Pathological angiogenesis	Proliferation Differentiation	Glycosylation [55] ADP-ribosylation [145]
GATAs	Vasculogenesis	Inflammatory response	Differentiation	ADP-ribosylation [146]
GAX			Differentiation	No data available
HAND	Vasculogenesis			No data available
HIF-1α	Angiogenesis	Pathological angiogenesis	Proliferation Differentiation Inflammation	ADP-ribosylation [147] Lactylation [107] Propionylation [148] S-nitrosylation [99]
ID	Angiogenesis	Pathological angiogenesis	Proliferation Differentiation	No data available
IRF-1		Inflammatory response		S-sulfhydration [85]
ISL2A	Vasculogenesis			No data available
JUNB	Angiogenesis		Differentiation	No data available
KLFs		Pathological angiogenesis Inflammatory response Vascular tone Endothelial-mesenchymal transition	Differentiation Proliferation Migration	S-sulfhydration [127]
LMO2	Vasculogenesis Angiogenesis			No data available
MAF	Angiogenesis			No data available
MECOM	Vasculogenesis Angiogenesis			No data available
MEF2	Angiogenesis	Pathological angiogenesis Endothelial-mesenchymal transition	Proliferation migration	Glycosylation [64] S-nitrosylation [123]
MSX1			Differentiation	No data available
MYOCD			Proliferation Differentiation Migration	No data available
NFAT	Angiogenesis	Pathological angiogenesis Inflammatory response Endothelial-mesenchymal transition	Differentiation Migration	Glycosylation [56] ADP-ribosylation
NKX2.5	Vasculogenesis	Endothelial-mesenchymal transition	Differentiation^e^	Glycosylation [65, 149]
NOTCH^b^	Angiogenesis	Pathological angiogenesis	Differentiation	Glycosylation [150]
NF-kB		Inflammatory response; Vascular Tone	Proliferation Differentiation Migration	Glycosylation [57] ADP-ribosylation [151] S-nitrosylation [101] S-sulfhydration [88]
NRF2	Angiogenesis	Vascular tone	Proliferation Differentiation	Glycation [70] ADP-ribosylation [152]
OCT3/4	Vasculogenesis	Endothelial-mesenchymal transition	Migration Differentiation	Glycosylation [153] Palmitoylation [154]
PAX9			Migration Differentiation	No data available
PPARγ		Inflammatory response Thrombosis Vascular tone	Differentiation Proliferation Migration	Glycosylation [66] S-nitrosylation [100] S-sulfhydration [86] Palmitoylation [155]
RUNX2			Proliferation differentiation	Glycosylation [58] Lactylation [49]
SMAD	Angiogenesis	Pathological angiogenesis	Proliferation Migration	Glycosylation [156] ADP-ribosylation [157] Palmitoylation [158]
SNAI 1	Angiogenesis	Endothelial-mesenchymal transition	Differentiation	ADP-ribosylation [159] Glycosylation [59] S-Nitrosylation [104]
SOXs	Angiogenesis	Pathological angiogenesis Inflammatory response		Glycosylation [67, 68] ADP-ribosylation [160] Lactylation [67, 161]
SP-1			Proliferation Differentiation	Glycosylation [162] S-sulfhydration [90]
STATs	Angiogenesis	Inflammatory response	Proliferation Differentiation	Glycosylation [69] ADP-ribosylation [163] Palmitoylation [164] Succinylation [165] S-sulfhydration [92]
TCF2			Proliferation Migration	No data available
TFEB	Angiogenesis	Inflammatory response	Proliferation Differentiation	Glycosylation [61] ADP-ribosylation [166] Lactylation [47]
TR3			Proliferation	No data available
TWIST		Endothelial-mesenchymal transition		No data available
VEZF1	Vasculogenesis Angiogenesis	Vascular tone		No data available
YAP^c^	Vasculogenesis Angiogenesis	Pathological angiogenesis Vascular tone	Proliferation Differentiation	Glycosylation [62] Lactylation [115]
YY1	Angiogenesis		Proliferation Migration	Glycosylation [63] ADP-ribosylation [167] Lactylation [116] S-nitrosylation [122]
ZEB		Endothelial-mesenchymal transition		Lactylation [168]

^a^The major roles of TFs are categorized in the indicated processes involving ECs and VSMCs during development and in adult life. However, it is not to be excluded overlapped fewer known activities. The quoted papers only refer in vivo data. Due to space limitation, references of listed TFs are omitted and available in specific reviews [169–171].

^b^NOTCH is not a TF, but its intracellular domain released after NOTCH activation by its cognate ligands translocates into the nucleus and forms a NOTCH transcription activation complex with RBPJ and MAML proteins.

^c^YAP is a transcriptional co-activator, which forms complexes with other TFs, including TEAD, SMADs, and T-box 5.

^d^PTMs based on acetylation, phosphorylation and methylation are not reported because almost characterizing all TFs.

^e^ The quoted references do not necessarily refer to PTM described in vascular cells.

^f^VSMC differentiation is generally characterized by the loose of contractile properties and the phenotypic transformation towards fibroblasts, chondrocytes, macrophages, adipocytes, osteoblasts, osteoclasts and lymphoid cells [169].

### Specific metabolic features of endothelial and vascular smooth muscle cells

While lipid and nucleotide metabolism in ECs and VSMCs are largely similar to those operating in other cell types, carbohydrate metabolism exhibits unique traits [32, 33]. ECs are among the human cells most exposed to oxygen, making them susceptible to damage caused by the production of reactive oxygen species (ROS). Therefore, the ECs metabolic setup is primarily geared towards minimizing this risk. ECs primarily generate ATP through glycolysis rather than oxidative phosphorylation. Indeed, a high NADH^+^ /NAD ratio in mitochondrial matrix and a high protonmotive force (the free energy required to move protons outward across a membrane) favors electrons leak and ROS generation [33]. This oxygen-independent ATP generation offers an additional advantage: when the endothelium contributes to tissue vascularization, it operates in an avascular environment with low oxygen concentrations, making glycolysis the most suitable pathway for energy production. Additionally, acetyl-CoA from fatty acid β-oxidation (FAO) enters the tricarboxylic acid (TCA) cycle to produce malic acid, which is exported into the cytosol and oxidized to pyruvate, generating NADPH^+^, which in turn supports glutathione production that contributes to redox homeostasis [34]. NADPH^+^ with ribose-5-phosphate is also produced by the pentose phosphate pathway (PPP), which has been reported to be crucial for EC activation [35]. An additional peculiar trait of EC metabolism relies on mitochondrial pathways. At least for the activation of the angiogenic program, TCA intermediates can mainly undergo cataplerotic reactions to sustain the biomass synthesis. To replenish the loss of TCA intermediates fueled by FAO, ECs show a prominent use of glutamine, which is converted to glutamate by a mitochondrial glutaminase and subsequently oxidized to alpha-ketoglutarate by a glutamate dehydrogenase. Therefore, the amount of ATP generated by oxidative phosphorylation is low and mainly used to sustain membrane ion transport [33]. A third specific feature is the production of nitric oxide (NO) involved in the control of vessels tone, permeability and in vasoprotection [33].

Similarly to endothelium, in VSMCs glycolysis accounts for about 90% of glucose metabolism under normoxia, resulting in a large production of lactate. Only 30% of ATP is produced by oxidative phosphorylation. Through the compartmentalization of energy requirements, the glycolytic ATP supports membrane potential maintenance, required for the effective depolarization process, while ATP generated by oxidative phosphorylation is utilized for cellular contractility mechanisms [32]. Lactate enhances mitochondrial reserve capacity, defined as the difference between the maximal respiratory capacity and the basal respiratory capacity. Lactate can indeed improve the efficiency of the electron transport chain by increasing pyruvate dehydrogenase (PDH) activity or by directly fueling the TCA cycle [36, 37]. As reported for ECs, the TCA cycle may also act in a cataplerotic manner by utilizing intermediates to generate biomass during VSMC proliferation.

**Fig. 3:**
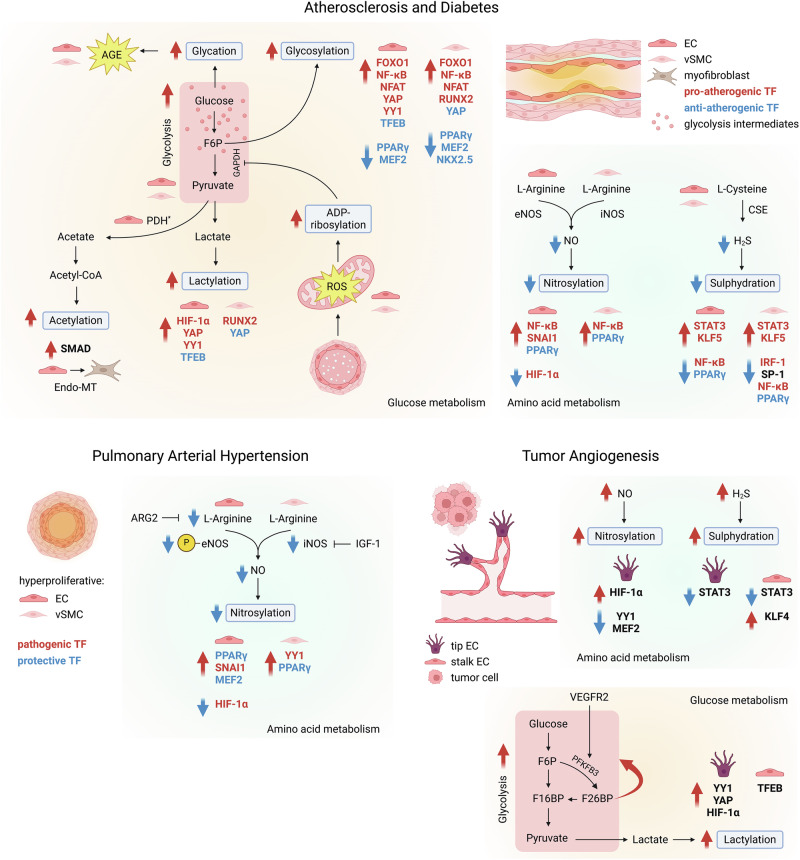
In vascular pathology, altered metabolic fluxes enhance or reduce metabolite-induced post-translational modifications (PTMs), which in turn, activate or inhibit the activity of transcription factors (TFs), participating in the disease pathogenesis. In atherosclerosis and diabetes (top panel), hyperglycemia causes reactive oxygen species production and DNA damage in mitochondria, leading to ADP-ribosylation and inhibition of GAPDH. The following block of glycolysis and the accumulation of its intermediates promotes glycosylation and lactylation of pro- and anti-atherogenic TFs, with consequential augmentation (red arrows) or diminishing (blue arrows) of their activity, both in endothelial and vascular smooth muscle cells. High glucose fuels glycation with the accumulation of advanced glycation end products (AGE), which amplify endothelial damage. The transforming growth factor-β (TGF-β)-induced alteration in pyruvate dehydrogenase activity in endothelial cells results in acetate production and SMAD acetylation, promoting endothelial-to-mesenchymal transition. The reduction in NO and H_2_S decreases TFs nitrosylation and sulphydration in both endothelial and vascular smooth muscle cells, with diverse outcomes. In tumor angiogenesis (bottom panel), vascular endothelial growth factor (VEGF) signaling enhances glycolysis, by increasing the activity of 6-phosphofructose-2-kinase-fructose-2,6-bisphosphatase (PFKFB3), which produces fructose-2,6-bisphosphate (F26BP), a positive allosteric effector of glycolysis. High rate of glycolysis, in particular, in tip cells, boosts lactylation and, therefore, activation of TFs responsible for tip phenotype maintenance. Increased NO and H_2_S production enhances TF nitrosylation and sulphydration with distinct effects in tip and stalk cells. Created with Biorender.com.

Here, we propose prospective and not necessarily exhaustive scenarios connecting metabolic hallmarks of paradigmatic vascular diseases and TF-PTMs (Fig. 3). As proof of concept, we will consider particular PTMs, such as lactylation, glycosylation, nitrosylation and sulfhydration, which respectively rely on glycolysis and amino acid metabolism.

### Atherosclerosis and diabetes

Atherosclerosis and diabetes develop as results of the alteration of lipid and carbohydrate metabolism, respectively, but both recognize the vascular unit as the earliest functional target. Furthermore, through hyperglycemia and insulin resistance, diabetes accelerates atherosclerosis progression by worsening the sterile inflammatory injury determined by lipoprotein accumulation in the vascular unit. ECs display a metabolic rewiring similar to what observed in cancer cells: most of the glucose-derived carbons are secreted as lactate and glutamine is the preferred carbon contributor to the TCA cycle.

#### Glycolysis

Altered glycolysis in ECs is a common hallmark of both atherosclerosis and diabetes. Modified shear stress at arterial bifurcations promotes glycolysis in ECs through the activation of mechano-transduction pathways orchestrated by YAP and inducing HIF-1α, both of which further drive glycolysis [38]. Similarly, VSMCs in atherosclerotic plaques display increased glycolytic flux [39]. In this case, enhanced glycolysis generally occurs during VSMC dedifferentiation induced by growth factors [40]. Increased lactate availability can facilitate lysine lactylation, a PTM that tends to stabilize target TFs, enhancing their activity. HIF-1α activity is precisely fine-tuned by several PTMs, principally phosphorylation and acetylation [41]. However in EC, it has been reported that also lactylation stabilizes HIF-1α under normoxia, potentiating the inflammatory response through NF-kB transcription [42]. The expression and activation of YAP and YY1 in the endothelium is triggered by shear stress alterations, and both TFs exert pro-atherogenic effects in vivo, which [43, 44] could be further enhanced by lactylation. Conversely, YAP lactylation in VSMCs may exert a protective effect, as YAP inhibits ferroptosis, an iron-dependent cell death mechanism involved in plaque instability [45]. Transcription factor EB (TFEB) is a TF involved in autophagy and lysosome biogenesis. TFEB activation is largely controlled by PTMs, including phosphorylation and acetylation [46]. It has been recently reported that, in cancer cells, TFEB lactylation prevents its ubiquitination and proteasome degradation, resulting in increased TFEB activity and autophagy flux [47]. Based on this information, we can postulate a link between glucose metabolism, the production of lactate, and the modulation of autophagy, through TF-PTM. Lactate-induced PTM on TFEB may mediate its stabilization and trigger sustained autophagy in other proliferating cell types, such as ECs or VSMCs, where glucose metabolism is reprogrammed and high concentrations of lactate are produced. Lactylation of endothelial TFEB could also have protective effects by mediating anti-inflammatory and anti-atherogenic actions [46, 47]. Another example is runt-related transcription factor (RUNX2), a master regulator of bone development, which drives the osteogenic differentiation in VSMCs, leading to plaque calcification process [48]. Interestingly, it has been shown in periodontal ligament stem cells that the osteogenic differentiation is promoted by RUNX2 lactylation, responsible for its stabilization through de-ubiquitination [49].

In ECs, increased glycolytic flux also favors endothelial-to-mesenchymal transition (EndMT) [50], a crucial pathophysiological process responsible for maintenance of chronic vascular inflammation and pathogenesis of several diseases, including atherosclerosis and PAH [51]. During EndMT, transforming growth factor-β (TGFβ) inhibits PDH activity, promoting acetyl-CoA production via acyl-coenzyme A synthetase short chain-2 (ACSS2), which leads to increased acetylation of SMAD transcriptional modulators perpetuating EndMT [52]. This suggests a critical role of acetate metabolism in the development of EndMT-related disorders, that may, at least in part, explain how hyperglycemia promotes arthrosclerosis.

In diabetes, hyperglycemia increases both cytosolic and mitochondrial ROS and promotes mitochondrial fixation, which are responsible for mitochondrial DNA damage in ECs [33]. DNA strand breaks activate poly (ADP-ribose) polymerase (PARP) resulting in ADP-ribosylation and inhibition of glyceraldehyde-3-phosphate dehydrogenase (GAPDH) [53]. As a result, the glycolytic flux is impaired with accumulation of intermediates. Among them, fructose-6-phosphate feeds hexosamine pathway leading to the production of amino sugars that sustain glycosylation reactions. Dihydroxyacetone phosphate and glyceraldehyde-3-phosphate can undergo spontaneous reactions to form methylglyoxal, which forms covalent adducts with arginine residues and DNA (glycation process) resulting in the synthesis of advanced glycation end products (AGE), which amplify endothelial damage [33].

The metabolic behavior of VSMCs under hyperglycemic stress mirrors that observed in ECs, with a glycolytic blockade induced by ROS, the accumulation of intermediates leading to the increased production of amino sugars and AGEs [32, 54]. However, unlike ECs, where hyperglycemia inhibits the PPP due to glucose-6-phosphate dehydrogenase blockade, the pathway is increased in VSMCs, providing antioxidant effects that partially attenuates the ROS-mediated injury [32]. This scenario is further exacerbated when hyperglycemia acts on VSMCs within atherosclerotic plaques, where the switch from a contractile to a synthetic phenotype further triggers glycolysis activation [32]. As a consequence, vascular unit cells accumulate the substrates available for glycosylation or glycation. It can be hypothesized that this process may target, among other proteins, TFs implicated in mechanisms that contribute to the development of the diseases.

Glycosylation enhances the transcriptional activity of the forkhead transcription factor FoxO1 [55], nuclear factor of activated T-cells NFAT [56], NF-κB [57], RUNX2 [58], SNAI1 [59], SP-1 [60], TFEB [61], YAP [62], and YY1 [63]; reduces that of MEF2 [64], NKX2.5 [65], PPARγ [66], SOX family members [67, 68] and stabilizes STAT5 [69]. Glycation, on the other hand, inhibits NRF2 [70]. FOXO1 respectively inhibits and enhances eNOS and inducible-NOS (iNOS), contributing to pro-atherogenic effects, as confirmed by the reduced progression of atherosclerosis in FOXO1-deleted mouse models [71, 72]. In VSMCs, insulin resistance promotes plaque instability by preventing FOXO1 activation [73]. Moreover, FOXO1 inhibits KLF2, a key anti-atherogenic regulator [74]. The pro-inflammatory and pro-atherogenic activities are positively regulated by NF-κB [75] and NFAT [76]. It is conceivable that glycosylation of YAP and YY1 further exacerbates vascular damage. YAP and YY1 are both activated by shear stress and are associated with increased endothelial proliferation, leading to higher permeability and promoting plaque formation. EC overexpression of a dominant-negative YY1 reduces atherosclerotic damage [43, 77]. By impairing transcriptional activity, glycosylation inhibits the protective effects of PPARγ, MEF2, and NKX2.5. PPARγ suppresses VSMC proliferation and endothelial involvement in inflammation [78]; MEF2 exerts anti-inflammatory effects and promotes KLF2 and KLF4 expression in ECs, both of which are anti-atherogenic and anti-inflammatory [79]; NKX2.5 stabilizes atherosclerotic plaques by inhibiting metalloprotease production and exerting anti-inflammatory effects [80]. Glycosylation of TFEB, on the other hand, may enhance its anti-atherogenic effect [46].

#### Arginine and cysteine metabolism

A good example of how deregulation of metabolite homeostasis can affect the PTMs of specific TFs involved in vascular biology is represented by the gasotransmitters hydrogen sulfide (H_2_S) and NO. In ECs and VSMC, H_2_S is generated from L-cysteine. The endogenous production of H_2_S is mainly mediated by cystathionine γ-lyase (CSE), which transforms cysteine to pyruvate, ammonia and H_2_S. H_2_S contributes to the vascular fitness by blocking vascular inflammation, exerting anti-oxidant effects, promoting vasorelaxation and inhibiting VSMCs proliferation (reviewed in [81]). Deficiency of CSE and the down-regulation of H_2_S lead to vascular disorders, such as atherosclerosis and hypertension [82]. Similarly, in diabetes models, H_2_S production in VSMCs is reduced concomitantly with the increase in proliferation [83]. Likewise, H_2_S donors and CSE inhibitors dampen and accelerate the atherosclerosis development, respectively [84]. H_2_S predominantly modifies its target proteins by sulfhydrating cysteine residues. *S*-Sulfhydration increases the activity of transcription repressor interferon regulatory factor 1 (IRF-1) [85], and the peroxisome proliferator activated receptor γ (PPARγ) [86]. H_2_S is an important mediator that exerts anti-atherogenic effects, inhibiting inflammatory response [87]. In physiological conditions, H_2_S induces DNA-binding activity of NF-kB, influencing the transcription of anti-apoptotic genes [8], while in pathophysiological conditions H_2_S interferes with the nuclear translocation of NF-kB and its binding to the promoter region of Monocyte Chemoattractant Protein 1 (MCP1), suggesting a pathophysiological role as anti-inflammatory system [89]. H_2_S increases *S*-Sulfhydration on specificity protein 1 (SP-1) in ECs [90] and cardiomyocytes [91]. In particular in ECs *S*-Sulfhydration stabilizes and prevents proteasome-mediated degradation of SP1 that mediates the transcription of vascular endothelial growth factor receptor 2 (VEGFR2) and neuroplin-1 and supports the vascular endothelial growth factor (VEGF)-induced proliferation and migration in ECs. In cardiomyocytes, H_2_S attenuates SP1 binding to Krüppel-Like factor 5 (KLF5) promoter, reducing KLF5 expression. H_2_S prevents elastin loss and the consequent phenotype switch and calcification by impairing STAT3 transcription activity in aortic smooth muscle cells [92]. SP-1 contributes to the phenotype switch from contractile to synthetic VSMCs by modulating metalloproteases [93]. However, other studies indicate that SP-1 inhibits VSMC proliferation [94]. Similar contradictory results have been reported for the pro- or anti-inflammatory role of SP-1 [95]. Activation of STAT3 and KLF5 facilitates the onset and progression of atherosclerosis by acting on both ECs and VSMCs. Active STAT3 and KLF5 promote VSMC proliferation and migration, and the acquisition of a pro-inflammatory phenotype in both ECs and VSMCs [96, 97]. Collectively H_2_S and related S-sulfhydration offer protection against several mechanisms involved in vascular dysfunction and maintaining vascular health. Therefore, is reasonable to assume a therapeutic application of H_2_S as donor in treatment of vascular dysfunctions like atherosclerosis and diabetes. Treatment with the H_2_S donor, NaHS, has been shown to significantly increase vessel growth [98]. Since reduced H_2_S production may possibly lower PTMs sulfhydration in vascular unit, a deeper understanding of the dynamic network of sulfhydration-susceptible TFs and the H_2_S concentrations required for this PTM could better clarify how H_2_S deficiency alters the transcriptional profile of the vascular unit in diabetes and atherosclerosis.

Arginine serves as a substrate for producing NO by nitric oxide synthases (eNOS, iNOS or nNOS). In the pathogenesis of atherosclerosis, ECs are dysfunctional and eNOS generates superoxide anion rather than NO by an electron leakage to molecular oxygen. On the contrary, NO produced by iNOS favors the contractile phenotype of the VSMCs. Alternatively, arginine is catabolized to ornithine and subsequently to polyamines, which support the synthetic phenotype characteristic of atherosclerotic lesions [38]. One of the mechanisms by which NO exerts its physiological and pathological functions is the S-nitrosylation, a reversible PTM that targets thiols at the cysteine residues, forming nitrosothiol (-SNO) groups. NO-mediated S-nitrosylation exerts different outcomes across TFs. S-nitrosylation can cause the stabilization and the activation of HIF-1α [99] or, conversely, can interfere with the DNA binding and suppress the transcriptional activity of PPARγ [100] and of NF-kB [101]. High levels of NO, induced by nitrosative stress, may compromise cellular functions by aberrant S-nitrosylation of TFs involved in vascular biology. For instance, reduced nitrosylation of HIF-1α and PPARγ could decrease their pro- [102] and anti-atherogenic activities [103], respectively, while NF-kB nitrosylation might enhance transcription of pro-inflammatory genes [75, 101]. Similarly, SNAI1 S-nitrosylation inhibits epithelial-mesenchymal transition [104], therefore it could counteract End-MT.

### Tumor angiogenesis

Tumor angiogenesis represents a paradigmatic example of pathological angiogenesis, a common hallmark of many chronic diseases [105]. Much of the current knowledge on the role of metabolic pathways stems from studies on sprouting angiogenesis [33]. Sprouting angiogenesis is characterized by the differentiation of ECs to a highly motile tip phenotype, responsible for guiding capillary plexus expansion, and to the stalk phenotype, which supports EC proliferation and lumen formation. Besides sprouting angiogenesis, other new blood vessel formation mechanisms have been described. Among these, vasculogenic mimicry is the formation of new blood vessels from cancer cells. Alternatively, tumor cells may position themselves adjacent to blood vessels (co-option mechanism). However, the cellular metabolism sustaining these mechanisms are unknown.

#### Glycolysis

Neoplastic tissues are characterized by acidosis due to high lactate levels produced by glycolysis (Warburg effect) and elevated glutamate concentrations stemming from glutamine metabolism. Therefore, tumor endothelium is characterized by a marked increase in glycolytic enzymes [33]. The VEGF/VEGFR2 pathway enhances the tip cell phenotype by increasing the activity of 6-phosphofructose-2-kinase-fructose-2,6-bisphosphatase (PFKFB3), which produces fructose-2,6-bisphosphate, a positive allosteric effector of glycolysis. Inhibition of PFKFB3 impairs both tip cell selection and stalk cell proliferation. Hypoxia is a feature of cancer associated to progression and resistance to therapies, triggering cellular stress and “glycolytic shift” through the activation of HIF-1α [106]. The high lactate availability in cancer drives lactylation of TFs, like HIF-1α, orchestrating tumor angiogenesis [107]. It is reasonable to predict that the increased lactate production represents a positive signal to potentiate angiogenic cues. However, the most significant modulatory effect of lactylation involves HIF’s regulation of angiogenic TF network. For example, HIF-1α activates FOXO1 in tip cells, and FOXO1 deletion blocks the tip phenotype [108]. FOXO1 acts as a gatekeeper for endothelial quiescence, reducing both glycolysis and mitochondrial respiration, thus playing a role in selecting tip cells and maintaining nearby ECs in a quiescent state [109]. HIF-1α is also able to promote the transcription of KLF4 [110] and ERG-1 [111]. KLF4 inhibits Notch ligands expression, promoting stalk phenotype [112]. Overexpression of KLF4 results in the activation and inhibition of VEGFA and NOTCH1 pathways, respectively [113]. ERG-1 plays a crucial role in promoting vascular development, maturation and stability [114].

In cancer, lactylation promotes the transcriptional activities of YAP [115] while TFEB lactylation at K91 prevents its proteasome degradation, resulting in high levels of autophagy [47]. Hypoxia-induced lactylation of YY1 in microglia leads to retinal neovascularization, through FGF2 transcription [116]. YY1 maintains low activity of NOTCH pathway allowing tip cell selection [117]. On the contrary, TFEB controls EC cycle and therefore it is presumably more important in stalk cell proliferation [118]. Therefore, it is reasonable to predict that the increased lactate production represents a positive signal to potentiate angiogenic cues. Of note, it is important to speculate that increased availability of lactate in growing tumor vessels might also derive from cancer cells as consequence of the Warburg effect. Lactate can be exported from cancer cells via monocarboxylate transporter (MCT) 4 and imported into ECs via MCT1 [119].

#### Arginine and cysteine metabolism

eNOS-derived NO is pivotal in regulating sprouting angiogenesis by controlling cell polarization to tip phenotype [120], thus, NO donors enhance EC proliferation and migration, whereas NOS inhibitors hinder these processes [121]. Likewise, EC functions related to angiogenesis are activated by H_2_S, and CSE blockade shows opposite effects [81]. Therefore, these gaseous signals might participate to S-nitrosylation and S-sulphydration of TFs regulating angiogenesis. The NO-induced S-nitrosylation of TFs involved in the angiogenesis process is context-dependent, exerting either angiogenic or antiangiogenic functions. Nitrosylation stabilizes HIF-1α [99] and increases its pro-angiogenic effects, but inhibits YY1 [122] and MEF2 [123]. As previously discussed, YY1 favors sprouting angiogenesis [124] and is a downstream effector of VEGFA pathway promoting Notch ligand DLL4 expression [125]. S-sulfhydration is a repressor of STAT-3 [92], a positive regulator of angiogenesis that promotes the expression of angiogenic inducers and metalloproteases [126]. H_2_S also prevents the degradation of KLF4, a regulator of endothelial barrier function and of tip-stalk dynamics [127]

### Pulmonary arterial hypertension

PAH is characterized by increased resistance in precapillary pulmonary vessels, leading to right heart failure. Early-stage EC apoptosis and neointima formation cause microvascular loss, while disease progression involves hyperproliferative ECs, smooth muscle cells, fibroblasts, and pericytes, promoting intraluminal obstruction and worsening pulmonary hypertension. The metabolic framework of the pulmonary vascular unit in PAH partially overlaps with that described for atherosclerosis and tumor angiogenesis. Alterations in metabolic pathways include shift to glycolysis, increased glutamine utilization and decreased fatty acid oxidation [128].

### Glycolysis

Glycolysis is significantly upregulated at the expense of oxidative phosphorylation in both pulmonary ECs and VSMCs [128]. This effect is more pronounced in the endothelium, where oxidative phosphorylation is typically more active, although still secondary to glycolysis in ATP production, as seen in other tissues. The key driver of this metabolic shift is HIF-1α. Mechanistically, this involves the increased expression or activity of glycolytic enzymes and glucose transporter GLUT1, alongside reduced oxidative phosphorylation complexes. The TCA cycle is reprogrammed, not for the generation of reducing equivalents but rather for the production of intermediates for biosynthesis [128]. As observed in diabetes and atherosclerosis, PAH is associated with oxidative damage. While not yet proven, it is likely that ROS inhibit GAPDH [52], as previously discussed for diabetes, leading to an accumulation of fructose-1,6-bisphosphate, fueling the hexosamine pathway and promoting glycosylation reactions. However, the role of this mechanism in contributing to glycosylation PTMs remains in early stages of investigation [129]. We propose that the metabolic alterations affecting PTMs (lactylation and glycosylation) of the TFs involved in both EC and VSMC functions are similar to those previously discussed for atherosclerosis and diabetes. This aligns with the pathogenic landscape of PAH, is characterized by enhanced angiogenesis, EndMT, and VSMC hyperproliferation. A specific effect of increased glycolysis in VSMCs is the stabilization of RUNX2 via lactylation [49], resulting in heightened HIF-1 activity, osteogenic differentiation of VSMCs, and promotion of vascular calcification and remodeling [130].

#### Arginine and cysteine metabolism

In PAH, endothelial NO production is diminished compared to normal pulmonary endothelium due to the inactivating phosphorylation of eNOS and reduced arginine availability, the latter being caused by increased mitochondrial arginase II activity. This reduced NO production is implicated in the disrupted balance between vasoconstrictive and vasodilatory molecules, contributing to pulmonary hypertension [128]. In VSMCs, NO bioavailability comes either from EC-derived NO or from iNOS activation, which can be induced by insulin-like growth factor 1 [128, 131]. Endothelial NO deficiency reduces its modulatory effects on key TFs involved in PAH, such as HIF-1α [116], PPARγ [84], SANIl [104] and MEF2 [123]. While HIF-1α exacerbates endothelial involvement and SNAIl promotes End-MT [132], PPARγ and MEF2 exert vasoprotective effects that counteract disease progression [133–135]. At the vascular medial layer, reduced NO bioavailability may alter the nitrosylation of PPARγ and YY1. PPARγ‘s protective role against PAH is evidenced by the fact that its deletion in VSMCs induces PAH [136]. In contrast, YY1 promotes the switch from a contractile to synthetic phenotype [137]. Current knowledge on the role of H_2_S in the pathogenesis of PAH is limited to data showing increased CSE activity in a hypoxia-induced PAH model [138], which was not confirmed in another study [139], and evidence that H_2_S donors exert a protective effect by inhibiting EndMT [140]. Therefore, it remains difficult to propose a clear hypothesis regarding the role of sulphydration PTMs in PAH.

## Conclusion remarks and future perspective

Based on data obtained from various cellular systems regarding the role of PTMs in modulating the function of TFs, this review outlines the effects of four prototype metabolite-induced PTMs (lactylation, glycosylation, nitrosylation, and sulfhydration) on the response of the vascular unit in diabetes, atherosclerosis, tumor angiogenesis and PAH. The proposed scenarios, while highly plausible, require robust validation and raise important questions.

We envisage that the interplay between metabolites and PTMs and the precise dosage of TFs are crucial for vascular plasticity and the pathophysiology of vascular diseases. It is likely that the regulation of TFs by PTMs maintains proper homeostasis in vascular unit, with metabolic shifts directly influencing TF activity and reprogramming transcriptional landscapes according to disease onset and progression. Understanding the molecular pathways governing metabolism-mediated PTM of TFs and programming the proper TF threshold may lead to novel therapeutic strategies aimed at restoring vascular integrity and function. The vascular system is primarily involved in cardiovascular diseases, either acquired or genetic in origin (e.g., lysosomal storage diseases, heritable cardiomyopathies, cardiac amyloidosis, vascular malformations, hereditary angioedema, ataxia-telangiectasia) and also contributes to the pathogenesis of numerous other conditions, including degenerative (cancer, nephropathies, autoimmune diseases, ocular diseases, neurodegenerative diseases, organ rejection), metabolic (obesity, diabetes, atherosclerosis), and infectious diseases. Therefore, transcriptional programs activated by the vascular unit in these conditions are potential druggable targets. For many years, TFs were considered poor pharmacological targets due to their pleiotropic effects. However, recent advances have deepened our understanding of DNA-TF-cofactor-enzyme complexes and how epigenetic mechanisms and PTMs regulate TF function. These insights enabled the design of specific inhibitors of transcriptional mechanisms, with several of these molecules already in clinical trials, particularly for hematologic malignancies.

The investigation of PTMs of TFs that require metabolites directly linked to perturbations in the cell metabolism is important in deciphering the functional and regulatory mechanisms in physiological and pathological processes involved in vascular biology. The field of PTMs of TFs is in its nascent stage, and many questions are still unanswered.
